# Annotations

**Published:** 1897-02-06

**Authors:** 


### ANNOTATIONS.

### Chilblains.

THE treatment of chilblains is at this time of year a matter of considerable interest, partly from the frequency of the complaint, partly from its obstinacy, and partly from the enormous number of remedies which have been vaunted for its cure. The great mass of these remedies consist of stimulating local applications of various kinds, of which it may be said they are nearly all more or less useful. It is notorious, however, not only that friction often fails to cure, but that in many cases chilblains occur while very active exercise is being taken, and pass away under a regimen in which such exercises take a far less prominent place. The fact is that chilblains do not occur in really healthy people. This is markedly the case with girls at school. Partly, no doubt, from want of exercise, but far more from want of fresh air, from monotony of diet, from want of regularity of the functions, and especially from a course of study for which they are by nature unfitted, their blood deteriorates, and when under such circumstances chilblains arise, the condition of health is a far more important factor in their production than mere "want of circulation." When chilblains vanish in the Christmas holidays it is as much the fresh air as the skating that does the good; it is as much the improvement of the condition of the blood as the mere driving of more blood through the injured part that is responsible for the cure. Professor Wright, of Netley, has recently pointed out that the particular condition of blood which is responsible for the occurrence of chilblains is one of deficient coagulability leading to serous effusion into the tissues. The treatment which he recommends is the administration of calcium chloride, the action of which is to increase the coagulability of the blood, and he has had considerable success. We incline, however, to the belief that more general measures than this will usually be desirable for the complete rehabilitation of the health; but anything which will relieve the chilblains themselves must be of great service in this direction, for nothing can more interfere than chilblains do with that outdoor life which is so desirable a means of curing the condition of which they are a symptom.

### The Hunger Whip.

"THE imperative nature of hunger," Carlyle tells us, "is well known." The value of hunger, the fact that it, or some equivalent of it, is indispensable in a world like ours, is by no means so universally recognised and admitted. A social experiment of considerable importance, which has for some time been tried in Switzerland, and which has just been abandoned, well illustrates the contention put forward by physiology, namely, that hunger is one of the most indispensable of all the forces in the human and animal worlds. The experiment consisted of insurance against want of employment. Two schemes were tried, one in the Canton of Berne, and one in St. Gall. The former was voluntary, the latter compulsory. The results in both cases are exceedingly instructive, and exceedingly futile. Or, rather, not futile, because they show how entirely mischievous non-natural economics may be. On the one hand, it has been found that a system which insured against loss of work promoted in a marked degree "laziness and idleness" among the shiftless; and on the other, the steady worker who "looked out" for employment for himself found himself handicapped and pecuniarily injured by the employment of "loafers" at higher wages than they were entitled to. Not only so, but in the case of St. Gall large numbers of unemployed labourers flocked in from other parts of Switzerland to share in the blessings of compulsory labour, and so to swamp those native to the district. Economic theorists abound in all civilised countries. It is eminently desirable that all of them should practise the methods in use among scientists in the study of their subjects: that is, they should try to discover how Nature works, and what her intentions may possibly be. The intention of Nature manifestly is that every grown man shall provide his own table; and if he finds himself in a set of circumstances where the table cannot easily be provided, or cannot be provided at all, then the command of Nature is to "move on," and look out for better circumstances elsewhere. In a word, Nature makes a man hungry; and, "the imperative nature of hunger being well known," the man discovers that it is less unpleasant to work than to starve. So he works. This is a great primitive force we are considering. It is a necessary force. The economist who interferes with it unduly is by no means wise. Even the Christian religion, the source of almost all our modern "pities," protests that, "if a man will not work," that is, will not try to keep himself in work, "neither shall he eat." When all is said and done, Nature is the sternest of all taskmistresses, and they are happiest who cheerfully recognise the fact. The whip of Nature hurts badly, but it is seldom used without real and undeniable necessity.

### Public-house Reform.

IN the persons of the Bishop of Chester and Professor Clifford Allbutt, M.D., of Cambridge, medicine and religion last week joined hands in the consideration of a question of deep importance from the standpoint of the health and well-being of the working classes. The Bishop, speaking at Cambridge, with Professor Clifford Allbutt presiding, advocated the making of experiments in the municipalisation of public-houses. Now the public-house question is exceedingly important from every point of view. No class of persons know so well as medical men the deadly effects of the drinking of too much alcohol, and especially of alcohol in crude, raw, and impure forms. If two things could be done for the working classes, namely, if the quantity of alcohol consumed by them could be reduced within healthy limits, and if the quality could be raised to a harmless level, the advantages resulting to the community would be incalculable. The tendency of the times is to *laisser faire* in this as in many other practical questions. But whatever state can afford to indulge the policy of *laisser faire*, no old state and no democratic state can. We ourselves must, as a nation, be up and doing, even though we sometimes do things which are less than wise. Why, then, may we not at least "try" the municipalisation of the drink business? If the plan succeeds, we can secure healthy instead of unhealthy public-houses, pure instead of impure drinks, efficient control of houses and sales; and, besides, we can put a large profit into the municipal pocket which now goes elsewhere. For many reasons we heartily wish well to the Bishop of Chester and Dr. Allbutt in their enterprise.

